# Successful staged surgery for advanced esophageal cancer after conversion pancreatoduodenectomy with pancreaticogastrostomy

**DOI:** 10.1007/s12328-025-02093-3

**Published:** 2025-01-14

**Authors:** Yuta Sato, Yoshihiro Tanaka, Yuji Hatanaka, Takeshi Horaguchi, Masahiro Fukada, Itaru Yasufuku, Ryuichi Asai, Jesse Yu Tajima, Katsutoshi Murase, Nobuhisa Matsuhashi

**Affiliations:** Department of Gastroenterological Surgery and Pediatric Surgery, Gifu University Graduate School of Medicine, 1-1 Yanagido, Gifu City, Gifu Prefecture 501-1194 Japan

**Keywords:** Esophageal cancer, Synchronous cancers, Staged surgery, Esophagectomy, Pancreatoduodenectomy

## Abstract

**Background:**

Complex surgery during initial cancer treatment can limit surgical options when planning management of a secondary malignancy. Subtotal esophagectomy and pancreatoduodenectomy are the most invasive and difficult procedures in gastroenterological surgery. Surgical cases in which subtotal esophagectomy was performed after pancreatoduodenectomy with pancreaticogastrostomy are extremely rare and challenging procedures due to the resulting complicated anatomical changes.

**Case presentation:**

A 60-year-old man with a history of conversion pancreatoduodenectomy with pancreaticogastrostomy for advanced pancreatic head cancer was diagnosed as having advanced thoracic esophageal squamous cell carcinoma. After neoadjuvant chemotherapy, we chose a two-staged surgery with thoracoscopic subtotal esophagectomy. Following percutaneous endoscopic gastrostomy, we performed subtotal esophagectomy, systematic lymph-node dissection, and esophagostomy as the first-stage operation. Fifty-six days later, we performed gastrointestinal reconstruction with pedicle jejunum and microvascular anastomosis by the percutaneous route as the second-stage operation. Postoperatively, the patient was relieved without major complications, and the tumors were amenable to curative pathologic resection.

**Conclusions:**

The greatest advantages of staged surgery are to reduce surgical invasiveness and to circumvent the lower rate of curability. Our procedure reported here may be recommended as an option for staged resection and reconstruction in patients with advanced esophageal cancer after pancreatoduodenectomy with pancreaticogastrostomy.

## Introduction

The number of reports of multiple primary cancers is currently increasing because of advancements in diagnostic imaging and technology [1], and the incidence of these cancers in patients with esophageal cancer is reportedly 5–36% [2–4]. The most frequently occurring multiple primary cancers are head and neck, gastric, and lung cancer, which is characterized by field cancerization [5]. However, with a frequency of 0.1–5%, double primary cancer of the esophagus and pancreas is rarely reported [6, 7]. Subtotal esophagectomy (SE) and pancreatoduodenectomy (PD) are widely considered the most invasive and difficult surgical procedures in gastrointestinal surgery. When both procedures are performed in one patient, it is expected to be particularly difficult to reconstruct the gastrointestinal tract due to anatomical changes and preserve the circulation of the reconstructed organs. In such cases, staged surgery can be a beneficial option to spread out the invasiveness to the patient [8, 9].

Herein, we report the successful performance of a two-staged surgery including oncological multidisciplinary treatment of advanced esophageal cancer in a patient who had previously undergone conversion PD with pancreaticogastrostomy for advanced pancreatic head cancer. We performed a very rare two-staged surgery as a video-assisted thoracoscopic SE and pedicle jejunum reconstruction with microvascular anastomosis.

## Case report

**Fig. 1:**
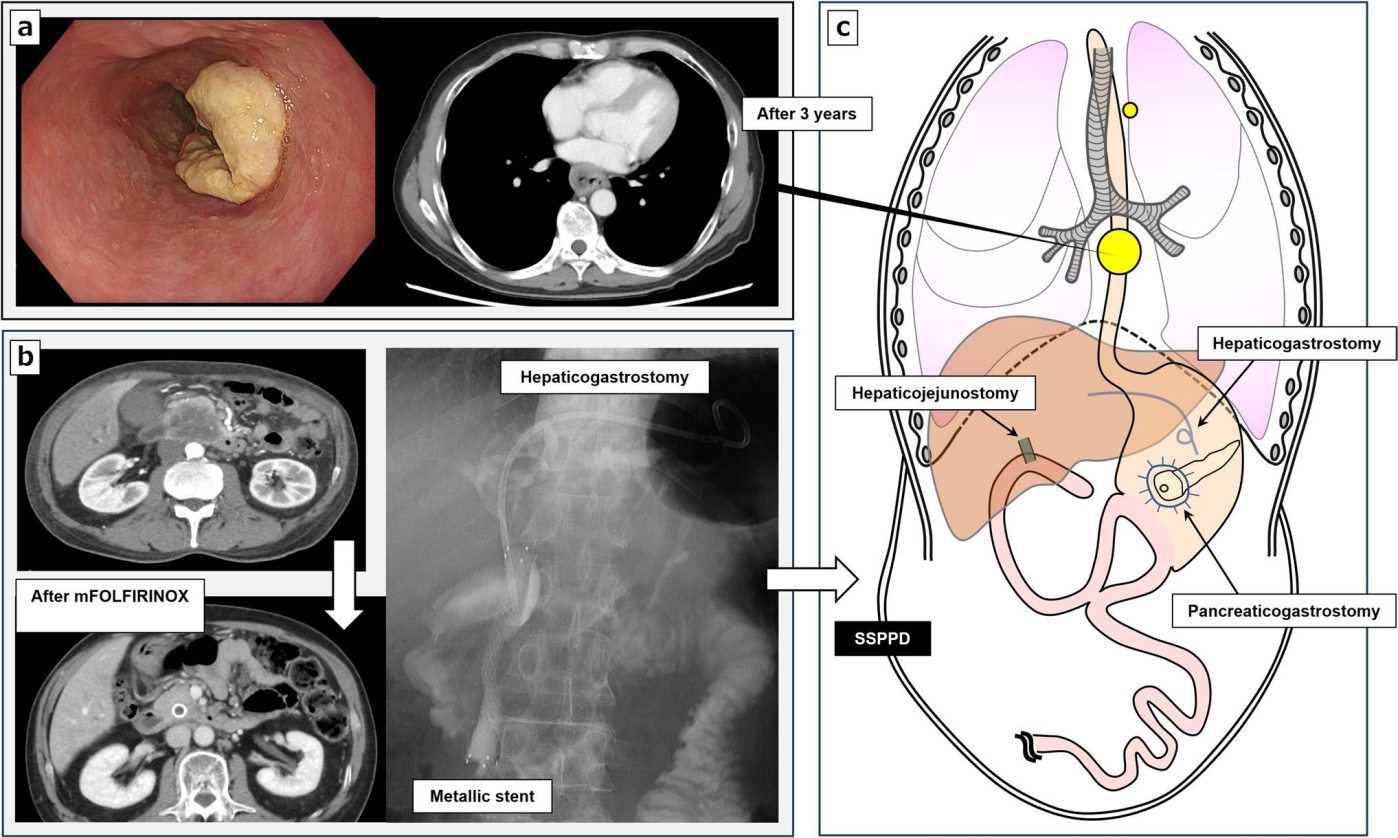
Schema showing the previous pancreatoduodenectomy and esophageal cancer. **a** Esophageal squamous cell carcinoma T3N1M0 in the middle thoracic esophagus. **b** Before and after chemotherapy with mFOLFIRINOX for pancreatic head cancer T4N0M0 with bile duct metallic stent and hepaticogastrostomy. **c** Schema of reconstruction after pancreatoduodenectomy with pancreaticogastrostomy prior to initiation of treatment for esophageal cancer. *SSPPD* subtotal stomach-preserving pancreatoduodenectomy

**Fig. 2:**
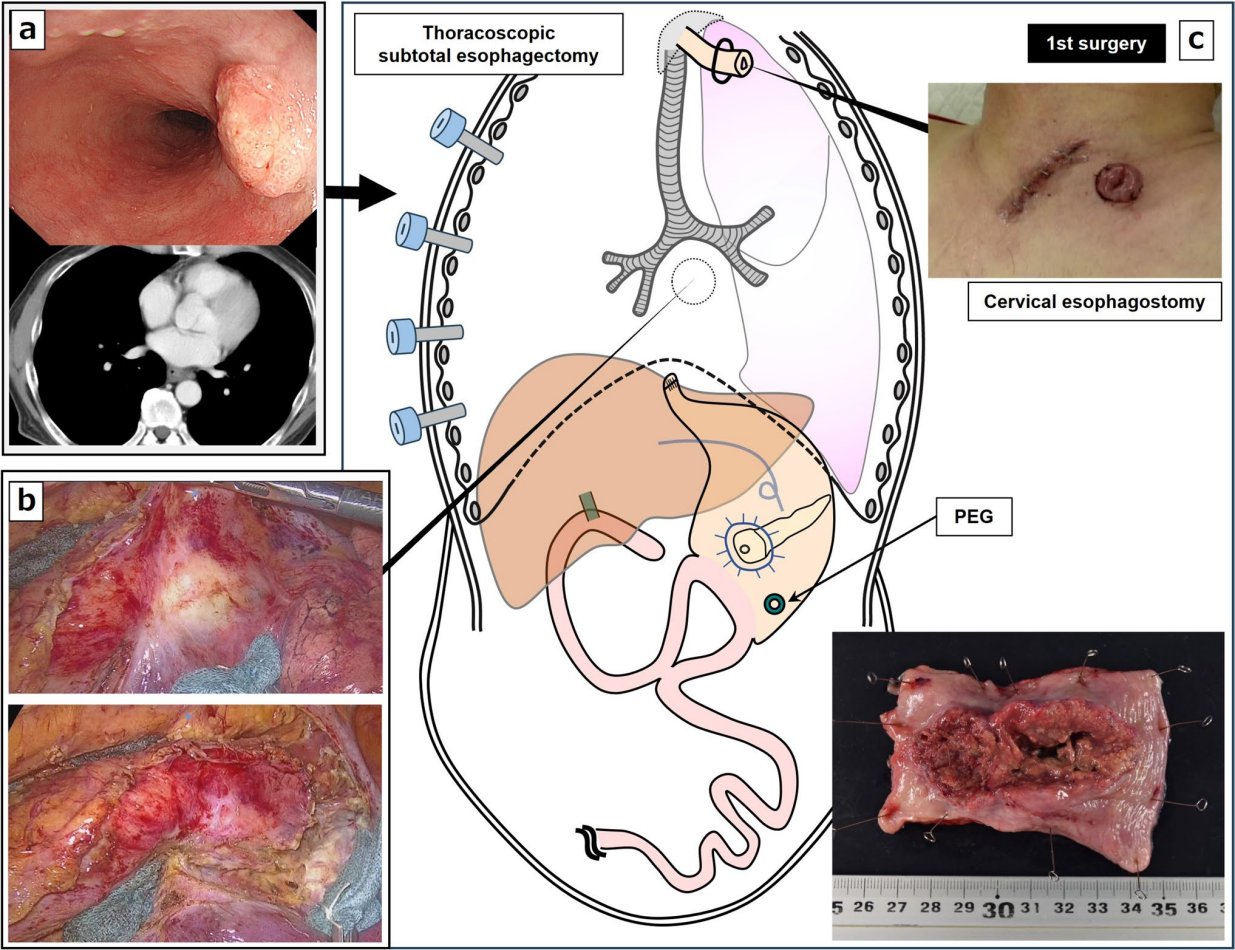
Findings after neoadjuvant chemotherapy for esophageal cancer and schema of the first surgery. **a** Esophageal cancer showed partial response. **b** Intraoperative findings. **c** Schema of the first surgery comprising subtotal esophagectomy and esophagostomy after percutaneous endoscopic gastrostomy (PEG) and surgical specimen of the esophagus

A 60-year-old man was referred from another hospital with a complaint of difficulty in swallowing food. He was diagnosed as having clinical stage III (T3, N1, M0) esophageal squamous cell carcinoma in the middle thoracic esophagus according to the TNM classification, 8^th^ edition (Fig. 1a). The patient had undergone a subtotal stomach-preserving PD (SSPPD) for advanced pancreatic cancer at our hospital 3 years earlier. He was diagnosed at age 57 to have clinical stage III (T4, N0, M0) pancreatic cancer in the pancreatic head according to the TNM classification, 8^th^ edition. He had severe obstructive jaundice, so a bile duct metallic stent was retained, and endoscopic ultrasonography-guided hepaticogastrostomy was done first. After about 7 months of anticancer drug treatment with modified folinic acid, 5-fluorouracil, irinotecan, and oxaliplatin (mFOLFIRINOX), the tumor had shrunk and become resectable with preservation of the superior mesenteric vessels (Fig. 1b). The patient then underwent SSPPD with pancreaticogastrostomy as a conversion surgery to avoid pancreatic fistula and postoperative bleeding for safety and long-term pancreatic duct patency (Fig. 1c). The operative time was 10 h 53 min, and blood loss was 2370 mL. He was discharged on postoperative day 22 with no complications. The pancreatic cancer was in the final stage III (T3, N0 [0/23], M0, Grade 2). Tegafur, gimeracil, and oteracil (TS-1) were administered as postoperative adjuvant chemotherapy for 6 months. Because the standard treatment for advanced esophageal cancer detected 3 years after SSPPD is preoperative chemotherapy [10], we first chose neoadjuvant chemotherapy (DCF: docetaxel 35 mg/m^2^, cisplatin 40 mg/m^2^, fluorouracil 400 mg/m^2^) [11, 12]. After two courses of neoadjuvant chemotherapy, the treatment efficacy was determined to be a partial response for the esophageal cancer (61% reduction) based on response evaluation criteria in Solid Tumors Criteria version 1.1 (Fig. 2a) [13].

Surgical planning raised some anatomical challenges. Ligation of the gastroduodenal artery was done for SSPPD, which gives rise to the right gastroepiploic artery, thus precluding the use of the stomach as conduit for any kind of esophagectomy. Extensive adhesiolysis could also be anticipated as the superior mesenteric vessels, which give rise to the middle colic vessels, were dissected during the SSPPD. Therefore, we chose a two-staged surgery to reduce surgical invasiveness and to circumvent the lower rate of curability. We planned esophagectomy and systematic lymph-node dissection as the first stage of the operation and gastrointestinal reconstruction with pedicle jejunum and microvascular anastomosis as the second stage of the operation. In the first operation after percutaneous endoscopic gastrostomy (PEG), the patient underwent thoracoscopic SE with mediastinal lymphadenectomy via prone thoracic manipulation. The esophageal cancer showed a tendency to invade the right inferior pulmonary vein, but R0 resection was possible (Fig. 2b). Subsequently, cervical manipulation was performed in the spine position, along with lymphadenectomy and neck cervical esophagostomy (Fig. 2c). The operative time was 5 h 15 min, and blood loss was 30 mL. The postoperative course was generally uneventful, and the patient was discharged home on the 21^st^ postoperative day.

**Fig. 3:**
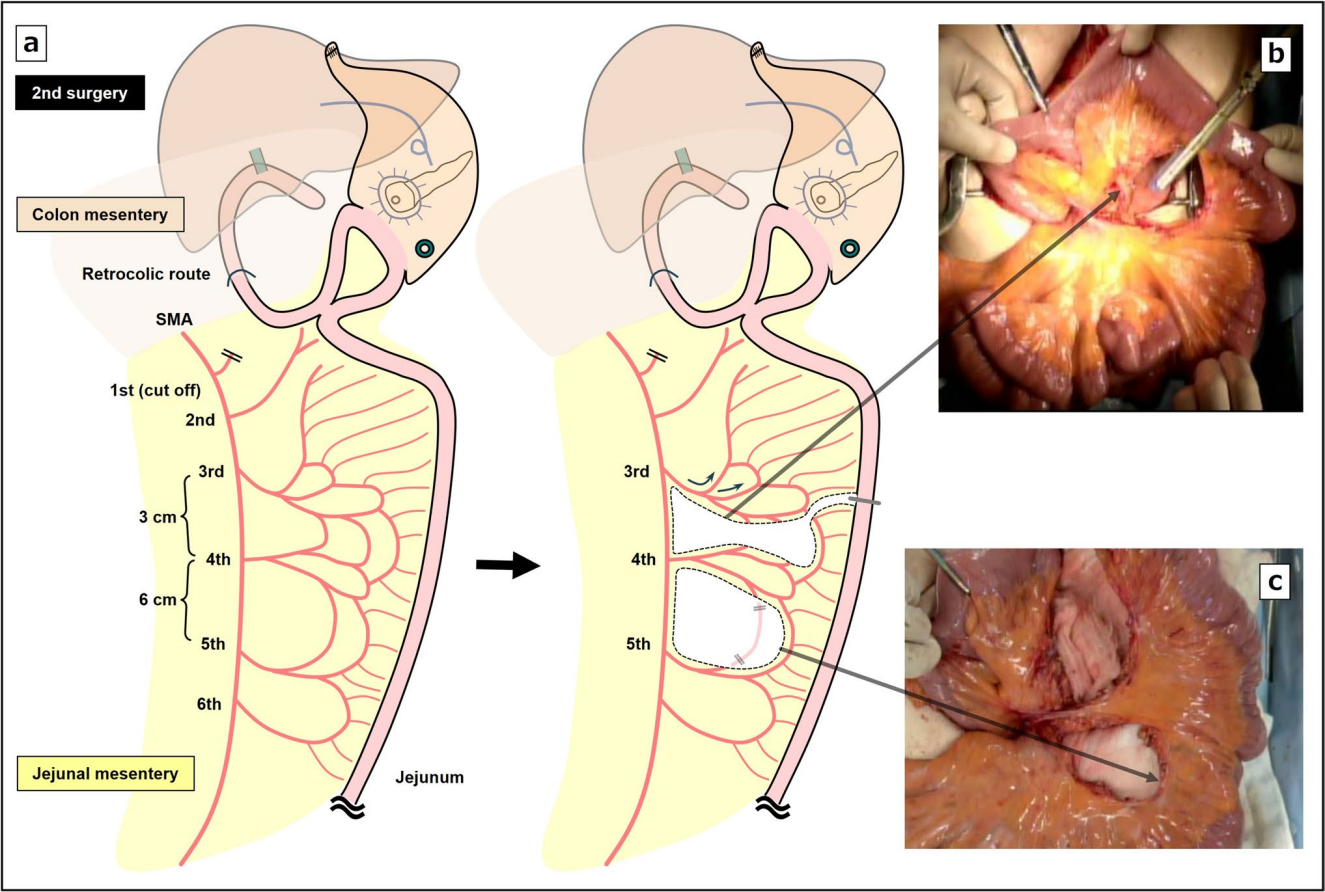
Schematic illustration and intraoperative photographs of the second surgery. **a** Processing of mesentery and creation of the afferent loop. **b** Vascular processing of the mesentery and creation of an elevated pedicle jejunum. **c** The incision of the mesentery was made as long as possible to extend the elevation distance

**Fig. 4:**
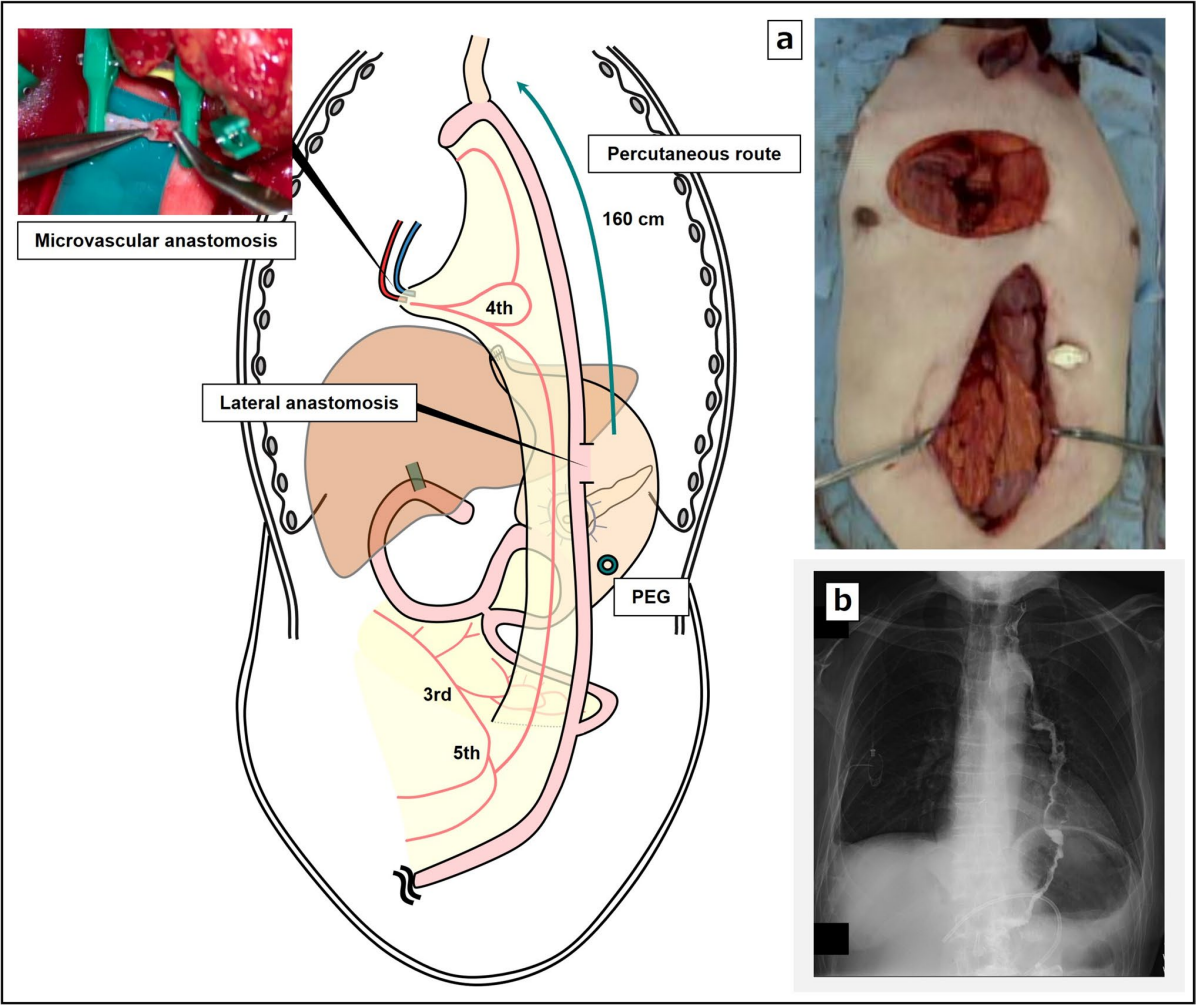
Surgical schematic illustrations after the second surgery. **a** The jejunum was elevated 160 cm by the percutaneous route, and the fourth jejunal artery and right internal thoracic artery were vaso-anastomosed. **b** Postoperative upper gastrointestinal angiography. *PEG* percutaneous endoscopic gastrostomy

Fifty-six days after the first-stage operation, the second-stage operation was performed. During abdominal manipulation via a median incision, extensive peritoneal adhesions were present. Abdominal lymph-node dissection was performed, followed by pedicle jejunal reconstruction. To ensure preservation of the circulation of the remnant stomach with pancreaticogastrostomy, the left gastroepiploic artery and short gastric artery were preserved, whereas abdominal lymphadenectomy was performed as usual for the patient’s thoracic esophageal cancer. When the mesentery of the jejunum was unfolded and observed, the first jejunal artery was found to have been transected during the PD, and the second jejunal artery flowed into the gastrojejunal anastomosis (Fig. 3a). During reconstruction of the pedicle jejunum, considering the blood flow in the afferent loop including the past gastrojejunal bypass and Braun’s anastomosis, the left branch of the third jejunal artery was used as a feeder to the afferent loop (Fig. 3b). The right branch was included on the side of the elevated jejunum, and the incision of the mesentery was made as long as possible by sacrificing one arcade to extend the elevation distance (Fig. 3c). An incision was made in the anterior chest and left neck to create the subcutaneous tunnel, the jejunum was elevated approximately 160 cm by the percutaneous route, and the fourth jejunal vein and the right internal thoracic vein were first anastomosed, and then, the fifth jejunal artery and the right internal thoracic artery were anastomosed (Fig. 4a). After additional resection of the cervical esophagus, an end-to-side anastomosis was performed between the esophagus and the elevated jejunum, as was a functional end-to-side anastomosis between the afferent loop and the elevated jejunum. In addition, a lateral anastomosis was made between the pedicle jejunum and the remnant stomach to allow a postoperative endoscopic approach to the stomach after surgery, and the hepaticogastrostomy tube was removed. The operative time was 8 h 55 min, and blood loss was 210 mL. The patient developed mild leakage at the esophageal jejunal anastomosis postoperatively, which quickly resolved with conservative treatment (Fig. 4b). The pathological result indicated esophageal squamous cell carcinoma, T3N0M0, final stage III, and the pathological response was Grade 1a. At 8 months after surgery, the patient remains recurrence free from both cancers. Postoperatively, nutritional support using the PEG was continued for 3 months, after which it was removed, because the patient was able to take adequate oral intake.

## Discussion

Multiple cancer disease refers to that in which more than one malignancy is diagnosed in the same patient, either simultaneously or sequentially. In patients with multiple cancers combined with esophageal cancer, a highly curative treatment such as that in the present case may be provided by choosing a staged operation, if necessary, in view of the operation time and degree of invasiveness. To achieve a better therapeutic effect, careful preoperative surgical planning is necessary, along with a multidisciplinary treatment plan that includes high-intensity preoperative chemotherapy and nutritional therapy [9, 14].

The overlap between malignant esophageal disease requiring SE and hepatobiliary pancreatic disease requiring PD is very rare. Any additional digestive surgeries, especially resectional procedures, performed after a prior SE or PD can be extremely difficult. The cases of simultaneous or metachronous SE and PD in the same patient are very interesting from both a scientific and a practical viewpoint. When divided chronologically, there are three possible clinical scenarios. The first is when the two diseases are noted simultaneously, the second is when PD is needed after SE, and the third is when SE is needed after PD. For the first clinical scenario, there are only 11 reported cases of esophageal and biliopancreatic tumors occurring simultaneously and requiring distal esophagectomy (DE) or SE and PD (Table 1). All of these cases required very long operative times and tended toward heavy bleeding, indicating that they are highly invasive procedures. When two malignant lesions are noted at the same time, as in this clinical scenario, it is possible to consider preoperatively whether to perform a simultaneous resection or a multi-staged resection, and there are multiple options for the treatment strategy. As shown in Table 1, 5 of the 11 cases were selected for two-staged surgery, and six underwent simultaneous resection. However, in almost all cases in which simultaneous resection was performed, DE was performed only via a transhiatal approach, and Ivor Lewis reconstruction was also performed [15–17]. In contrast, staged surgery tended to be chosen for cases requiring a more invasive thoracic approach, such as SE and McKeown esophagectomy [18–21]. Of the five patients who underwent staged surgery, SE was performed in the first surgery and PD in the second surgery in four of these patients. In some cases, the reason for choosing this sequence is that the order is determined by factors that affect the degree of tumor progression and prognosis [22]. There are also reports that SE was performed first due to concerns about postoperative complications, especially pancreatic fistula after PD [19]. Another advantage of splitting the thoracic and abdominal manipulations into two-staged surgery is that the first operation can focus on tumor resection and lymph-node dissection, whereas the second operation focuses on gastrointestinal reconstruction along with abdominal dissection [20].

**Table 1:** Case series in which esophagectomy and pancreatoduodenectomy were necessary due to synchronous malignancies

Author	Year	Age	Sex	Diagnosis	Stage (interval)	Approach for esophagectomy	Resection	Reconstructed organs and routes	Operation time blood loss	Complication
Mafune	1995	64	M	- ESCC - AVA (after distal gastrectomy)	One	Right thoracotomy	SE, PD (child) total residual gastrectomy	Ileocolon (percutaneous)	960 min 3120 ml	Leakage at esophagoileostomy
Kurosaki	2000	72	M	- ESCC - IPMN	One	Transhiatal	DE, PPPD (cattle)	Gastric tube (retrosternal)	N/A	No complication
Jayaprakash	2009	62	M	- EGJ adenocarcinoma - AVA	One	Transhiatal	DE, PD (child), total gastrectomy	Pedicle jejunum	340 min 620 ml	No complication
Gyorki	2011	58	M	- EGJ adenocarcinoma - Pancreatic NEC	Two (6 months)	Right thoracotomy	1) SE 2) PPPD (child)	Right hemicolon	1) 510 min 2) 335 min	DGE
Kim	2011	65	M	- EGJ adenocarcinoma - Pancreatic head cancer	One	Transhiatal	DE, SSPPD	Jejunal interposition	960 min 1000 ml	Atrial fibrillation
Fukaya	2014	69	M	- ESCC - AVA - Early gastric cancer	Two (35 days)	Right thoracotomy	1) SE, esophagostomy, gastrostomy 2) PD (child), total gastrectomy	Ileocolon (percutaneous)	1) 516 min 374 ml 2) 752 min 1900 ml	Pancreatic fistula
Fukaya	2016	68	M	- ESCC - Local recurrence of AVA	Two (40 days)	Right thoracotomy	1) SE, esophagostomy, gastrostomy 2) PPPD (child)	Gastric tube (percutaneous)	N/A	Pancreatic fistula, leakage at esophagogastrostomy
Rosati	2017	72	M	- Esophageal metastasis - Pancreatic ductal carcinoma	One	Transhiatal	DE, PPPD	Gastric tube (Ivor Lewis)	N/A	SSI
Ozawa	2019	70	F	- ESCC - Pancreatic head cancer	Two (28 days)	Right thoracoscopic	1) SE, PEG, esophagostomy 2) PD (child), total gastrectomy	Ileocolon (percutaneous)	1) 490 min 100 ml 2) 611 min 724 ml	1) Recurrent laryngeal nerve paralysis 2) Leakage at esophagoileostomy
de la Vega	2021	77	M	- EGJ adenocarcinoma - AVA	One	Transthoracic	DE, SSPPD (whipple), total gastrectomy	Right hemicolon	N/A	Jejunal perforation (reoperation)
Studier-Fischer	2023	67	M	- EGJ adenocarcinoma - Multiple pancreatic lesion (metastasis of RCC)	Two (5 days)	Right thoracotomy	1) Total PD, gastrostomy 2) DE, total gastrectomy	Transverse colon (Ivor Lewis)	1) 1800 ml 2) N/A	No complication

*ESCC* esophageal squamous cell carcinoma, *AVA* ampulla of Vater adenocarcinoma, *SE* subtotal esophagectomy, *DE* distal esophagectomy, *PD* pancreatoduodenectomy, *PPPD* pylorus-preserving PD, *SSPPD* subtotal stomach-preserving PD, *IPMN* intraductal papillary mucinous neoplasm, *EGJ* esophagogastric junction, *NEC* Neuroendocrine Carcinoma, *DGE* delayed gastric emptying, *PEG* percutaneous endoscopic gastrostomy, *RCC* renal cell carcinoma, *SSI* surgical site infection, *N/A* not applicable

In the second and third clinical scenarios, the need for SE and PD occurs heterochronically. The treatment strategy is limited by the need for atypical procedures with respect to lymph-node dissection and reconstruction methods, because these two surgical interventions significantly alter the normal anatomical relationship of the upper abdomen. The second clinical scenario, in which PD is required after SE, is not rare, and a relatively large number of reported cases are scattered throughout the literature [23–26]. As shown in Table 1, in almost all cases of staged resection for simultaneous cancers, the sequence of SE first, followed by PD, was chosen in almost all cases, which confirms that this surgical sequence is not impossible. However, the third clinical scenario, for which disease requiring SE was noted after PD surgery, was reported extremely infrequently, with only five cases, including our case and one case of benign disease (Table 2) [27]. Only three cases of SE required cervical gastrointestinal anastomosis, which is more invasive than DE, including the present case, and all were limited to 2019 or later [28, 29]. Two important oncological points are worth mentioning in our case. One is that both pancreatic head and esophageal cancers were highly advanced, and high-intensity chemotherapy, such as mFOLFIRINOX, TS-1, and DCF, were used in the perioperative period for each disease, with the earlier PD being a conversion surgery, whereas radical lymph-node dissection was required for the later SE. The other is that the PD reconstruction procedure performed prior to SE was a pancreaticogastrostomy. This has never been reported before to our knowledge. If the esophageal cancer is an early stage cancer, as reported by Morikawe et al. [29], one-stage reconstruction with SE using robotic surgery may be possible, or a single resection and reconstruction may be possible for DE for esophagogastric junction cancer [30]. If the cancer is advanced, as in the present case, and long-term chemotherapy is required, we believe that choosing a two-staged surgery will improve oncologic safety. Because of the shortage of jejunum available for reconstruction due to Child’s reconstruction for PD, the right hemicolon is generally supposed to be suitable for esophageal reconstruction. A colon graft can easily be brought up to the neck without microvascular anastomosis, making it a favorable procedure [31]. However, jejunal interposition offers functional advantages, because it more closely resembles the esophagus, and the versatility of the jejunal flap is useful in solving various complex scenarios [16]. In addition, in our presented case, extensive colonic adhesions near the hepatic and splenic flexures complicated the colon reconstruction.

**Table 2:** Case series of esophagectomy after pancreatoduodenectomy

Author	Year	Age	Sex	Disease (requires PD)	Reconstruction	Period after PD	Disease (requires SE or DE)	Staged (interval)	Approach	Resection	Reconstructed organs and routes
Dellaportas	2016	69	M	Pancreatic head cancer	PPPD (whipple)	3 years	EGJ adenocarcinoma	One	laparotomy	DE, total gastrectomy	Pedicle jejunum Ivor Lewis
Fukaya	2016	63	M	Bile duct carcinoma	PPPD (child)	158 days	Esophageal stricture	One	Laparotomy, thoracotomy	DE, total gastrectomy	Transverse colon Ivor Lewis
Asai	2019	71	F	AVA	SSPPD (child)	10 years	Metastasis of Brest cancer	Two (61 days)	Thoracoscopic	SE	Pedicle jejunum Percutaneous
Moriwake	2024	73	M	IPMN	PPPD (child)	3 years	ESCC	one	Thoracoscopic (robot)	SE	Free jejunum Percutaneous
Our case	2024		M	Pancreatic head cancer	SSPPD (pancreaticogastrostomy)	3 years	ESCC	Two (56 days)	Thoracoscopic	SE	Pedicle jejunum Percutaneous

*SE* subtotal esophagectomy; *DE* distal esophagectomy; *PD* pancreatoduodenectomy; *PPPD* pylorus-preserving pancreatoduodenectomy; *SSPPD* subtotal stomach-preserving pancreaticoduodenectomy; *EGJ* esophagogastric junction; *ESCC* esophageal squamous cell carcinoma; *AVA* ampulla of Vater adenocarcinoma; *IPMN* intraductal papillary mucinous neoplasm

To the best of our knowledge, this is the first report to describe minimally invasive SE for advanced esophageal squamous cell carcinoma after SSPPD with pancreaticogastrostomy for advanced pancreatic head cancer. The decision on surgical technique in this patient was very difficult, and we planned a two-staged surgery after a thorough preoperative review. In conclusion, the procedure reported here may be recommended as an option for staged resection and reconstruction in patients with simultaneous advanced esophageal cancer after PD. The use of complex surgery in the treatment of cancer patients has made reoperations challenging. However, staged surgery options exist, and meticulous preoperative planning and intraoperative judgment can help the surgeon to perform an extensive and successful oncologically sound procedure.

## Abbreviations

SE: Subtotal esophagectomy
PD: Pancreatoduodenectomy
SSPPD: Subtotal stomach-preserving pancreatoduodenectomy
mFOLFIRINOX: Modified folinic acid, 5-fluorouracil, irinotecan, and oxaliplatin
TS-1: Tegafur, gimeracil, and oteracil
DCF: Docetaxel, cisplatin, and fluorouracil
PEG: Percutaneous endoscopic gastrostomy
DE: Distal esophagectomy
